# Physico-Chemical and Electrochemical Properties of Nanoparticulate NiO/C Composites for High Performance Lithium and Sodium Ion Battery Anodes

**DOI:** 10.3390/nano7120423

**Published:** 2017-12-02

**Authors:** Amaia Iturrondobeitia, Aintzane Goñi, Izaskun Gil de Muro, Luis Lezama, Teófilo Rojo

**Affiliations:** 1 Departamento de Química Inorgánica, Universidad del País Vasco UPV/EHU, P.O. Box 644, 48080 Bilbao, Spain; amaia.iturrondobeitia@ehu.eus (A.I.); aintzane.goni@ehu.eus (A.G.); izaskun.gildemuro@ehu.eus (I.G.d.M.); trojo@cicenergigune.com (T.R.); 2 BCMATERIALS, Ibaizabal Bidea 500, Parque Científico y Tecnológico de Bizkaia, 48160 Derio, Spain; 3 CIC energiGUNE, Parque Tecnolóogico de Álava, Albert Einstein 48, 01510 Miñano, Álava, Spain

**Keywords:** nanoparticulate NiO, lithium ion batteries, sodium ion batteries

## Abstract

Nanoparticulate NiO and NiO/C composites with different carbon proportions have been prepared for anode application in lithium and sodium ion batteries. Structural characterization demonstrated the presence of metallic Ni in the composites. Morphological study revealed that the NiO and Ni nanoparticles were well dispersed in the matrix of amorphous carbon. The electrochemical study showed that the lithium ion batteries (LIBs), containing composites with carbon, have promising electrochemical performances, delivering specific discharge capacities of 550 mAh/g after operating for 100 cycles at 1C. These excellent results could be explained by the homogeneity of particle size and structure, as well as the uniform distribution of NiO/Ni nanoparticles in the in situ generated amorphous carbon matrix. On the other hand, the sodium ion battery (NIB) with the NiO/C composite revealed a poor cycling stability. Post-mortem analyses revealed that this fact could be ascribed to the absence of a stable Solid Electrolyte Interface (SEI) or passivation layer upon cycling.

## 1. Introduction

As one of the most important and widely used rechargeable power sources, lithium ion batteries (LIBs) have been widely used in portable electronics, electric vehicles (EVs), and hybrid electric vehicles (HEVs) [1,2,3,4]. Additionally, they are supposed to be one of the most promising candidates for next generation power sources. Besides of LIBs, recently, sodium ion batteries (NIBs) have received increased attention as an alternative to LIBs for stationary storage due to the bundance and low cost of Na. Actually, NIBs were initially studied when the development of LIBs began in the 1970s, but due to the fast advances in the development of LIBs, NIBs were unregarded [5]. Even if the fundamental principles of the NIBs and LIBs are almost the same, NIBs usually exhibit low specific capacities, short cycle lifes, and poor rate capabilities due to increased radius and mass of Na (1.02 Å, 22.99 g/mol) when compared to that of Li (0.59 Å, 6.94 g/mol) [6]. Additionally, sodium has a higher standard electrode potential as compared to lithium (−2.71 V vs. Standard hydrogen electrode (SHE) as compared to −3.02 V vs. SHE for lithium). Consequently, NIBs will often fall short in terms of energy [7]. Nevertheless, the weight of cyclable lithium and sodium is only a small part of the mass of the components of the electrode.

Nowadays, even if graphite is the most widely used anode material due to its low cost, high abundance, and outstanding electrochemical performance, this material exhibits a theoretical capacity of 372 mAh/g. Consequently, in order to fulfill the requirements as to large scale applications, higher energy density systems need to be developed. This purpose implies the necessity of denser and higher capacity anode materials are needed. 

In this sense, three-dimensional (3D) transition metal oxides (MO_x_) are among one of the most promising next-generation anode materials under consideration due to their low cost, high theoretical capacities (500–1000 mAh/g) and easy fabrication [8,9]. MO_X_ anodes can be classified into three different groups depending on their reaction mechanisms: (a) Li alloy reaction mechanisms, (b) insertion/extraction reaction mechanisms, and (c) conversion reaction mechanisms. In this regard, most of the MO_x_ materials present conversion reactions vs. Li based in the formation and decomposition of Li_2_O as shown below [10,11]:

M_x_O_y_ + 2yLi^+^ + 2ye^−^ ↔ xM + yLi_2_O

The transition metal oxides are ultimately reduced to the elements leading to composite materials consisting of metallic nanoparticles dispersed in an amorphous Li_2_O matrix. The formation of metallic nanoparticles is a key factor when increasing the reversibility of the reaction. In this regard, the reactivity of the nanoparticles is increased in the Li_2_O matrix in which they are embedded due to the high surface area of the nanoparticles. However, the main drawback of these MO_x_ materials for their application in LIBs as anode materials is the large irreversible capacity that they suffer in the first cycle. Nevertheless, this irreversible capacity can be successfully addressed by surface prelitiation treatments or by thin film nano-structuration [12,13].

NiO has been regarded as one of the most popular choices of metal oxides due to its high theoretical capacity (718 mAh/g), high corrosion resistance, and low materials and processing costs [14]. However, further optimization of nickel oxides as anode materials is needed due to their poor capacity retention or rate capability that is owed to low electric conductivity and large volume change during the conversion reaction [15,16]. Many attempts have been made to improve the reversible capacity, cycling stability, and rate capability of NiO. Towards this end, the strategies of introducing carbonaceous materials, such as grapheme [17], and nanoestructurating the material [18] have shown enhanced capacities. In this regard, carbonaceous materials, as well as increasing the electronic contact between the iron oxide particles, also buffer the volume and structural changes associated with the transformation of iron oxide particles into metallic iron during the discharge/charge process.

Even if transition metal oxides have been extensively studied in LIBs, only a few metal oxides have been studied for application in NIBs [19,20]. Among these studies, some previous reports have demonstrated the potential application of NiO in NIBs [21]. Meanwhile, other researchers have revealed the electrochemical inactivity of NiO with Na, while exhibiting outstanding performances in LIBs. In this regard, the reason why this is happening is not yet clearly understood [22]. As far as we are aware, very little research has been done in the field of NiO anodes for NIBs application up to now.

Herein, we follow the double strategy of nanostructurating the material and introducing a source of carbon so as to make composite materials in order to overcome some of the problems concerning the application of nickel oxide as anode for batteries. Most of the preparation methods of high-performance nickel oxides are excessively complicated besides of being of low yield. Nevertheless, in this work, a freeze-drying synthesis procedure has been employed, which as well as being a cheap and easy method, can be easily implemented as part of an industrial process. Additionally, the freeze-drying method offers a uniform homogeneity of reactants, the possibility of introducing a carbon source, and the advantage of using lower calcination temperatures [23]. The efficiency of this synthesis method that allows for obtaining nanosized cathodic materials with exceptional electrochemical performance has already been demonstrated in several studies [24]. 

In this study, three different composites based on nanosized NiO and carbon, were successfully synthesized by the freeze-drying method. We report on the structural, morphologic, magnetic, spectroscopic, and electrochemical characterization (vs. Li and Na) of the synthesized samples, establishing correlations among the composition, morphology, and electrochemical performance. Particular attention has been paid to the post-mortem analysis of NIBs in order to understand why the same material behaves differently when applied as anode for LIBs and NIBs.

## 2. Results and Discussion

The amount of carbon of the three NiO/C samples synthesized by freeze-drying method was determined by elemental analysis. Table 1 summarizes the percentage of C in each sample. Accordingly, the samples were called NiO_18%C, NiO_29%C and NiO_air as this material was calcined in air. 

**Figure 1:**
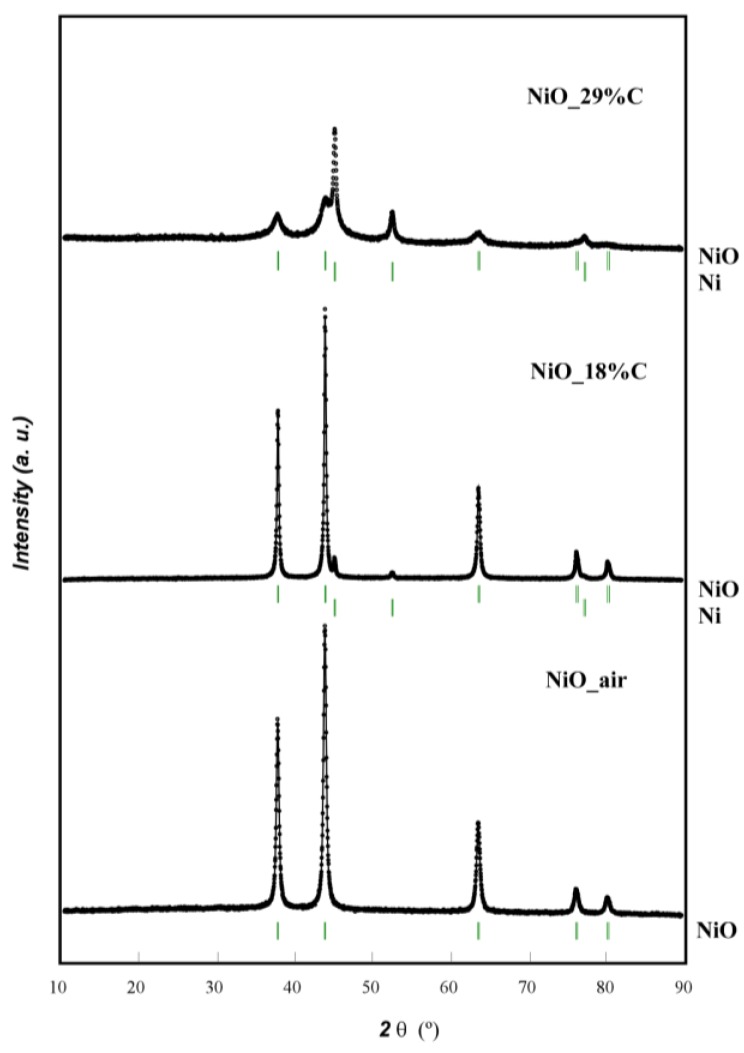
XRD patterns of NiO_air, NiO_18%C and NiO_29%C.

Figure 1 shows the X-ray diffraction (XRD) patterns of NiO_air and the two composites. For the NiO_air sample, all of the diffraction peaks could be indexed to pure phase cubic nickel oxide (Powder Diffraction File 78-0429 PDF card). No additional reflections were detected indicating the absence of impurities. In the case of NiO_18%C, two weak reflections can be detected at 2θ ≈ 45° and 53° corresponding to metallic nickel (Powder Diffraction File 88-2326 PDF card). However, different from NiO_air and NiO_18%C samples, the diffraction maxima of NiO_29%C composite appears to have less intensity and higher broadening. Additionally, the reflections corresponding to metallic nickel have higher intensity in this sample than in the former ones. This could be attributed to the higher amount of carbon in this sample, as it probably has led to a more reducing atmosphere and consequently, a higher amount of Ni (II) has been reduced to Ni (0) [25]. It is remarkable that the in situ generated carbon cannot be detected in any of the cases due to its amorphous character. The theoretical and experimental cell parameters obtained from profile-fittings are shown in Table 2.

**Figure 2:**
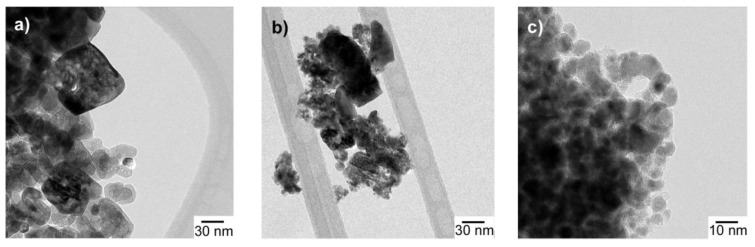
TEM images of (**a**) NiO_air, (**b**) NiO_18%C, and (**c**) NiO_29%C samples.

Preliminary SEM images (Supplementary information SI.1) allowed for asserting that the NiO_air sample is composed of irregularly shaped particles with a wide range of size (5–50 nm). In the same way, NiO_18%C and NiO_29%C composites seemed to contain nanoparticles homogeneously dispersed in the in situ generated carbon matrix. In order to further investigate that morphology, TEM measurements were carried out. Figure 2a–c shows transmission electron micrographs of the NiO_air, NiO_18%C, and NiO_29%C samples, respectively. The NiO_air sample has a heterogeneous aspect and it is made up of irregularly shaped particles with a wide range of sizes (5–50 nm). Apparently, the bigger particles show a porous morphology, which could probably be attributed to the formation of gas bubbles during the combustion in air of the organic reagents that are present in the synthesis process. The micrograph of the NiO_18%C sample, Figure 2b, shows that the sample is constituted by nanometric size particles ranging in diameter from 10 to 20 nm. Additionally, the sample shows quite a heterogeneous appearance and is difficult to clearly visualize the in situ generated carbon matrix. The NiO_29%C composite, Figure 2c, is made up of 5–10 nm homogeneous spherical nanoparticles that are embedded in the in situ generated carbon matrix. The particle size of NiO_29%C sample was the smallest of all the samples as the high amount of carbon in this composite acted preventing the growth of particle size.

The magnetic hysteresis loops at room temperature of the NiO_air, NiO_18%C, and NiO_29%C samples allowed to calculate the amount of metallic nickel in each sample (Supplementary information SI.2). The NiO_air sample shows an almost a null magnetization for the whole range of the applied magnetic fields. This fact could be assigned to the completely antiferromagnetic ordering of NiO phase below its Neel temperature (T_N_ = 525 K). In the case of NiO_18%C and NiO_29%C samples, the magnetization shows a clear dependence in the presence of the applied magnetic field. The magnetic response to the external field matches with the presence of the ferromagnetic compound Ni (0) in the samples, which it is Curie temperature (TC = 631 K) is well above of the experiment temperature. 

The magnitude of the saturation magnetization in these samples is just influenced by the percentage of metallic nickel. In this sense, it is straightforward to estimate the percentage of metallic nickel in each sample by assuming a saturation magnetization value of 55 emu/g [26] for Ni (0). Table 3 gathers the values of the saturation magnetization and the percentages of metallic nickel for each sample.

**Figure 3:**
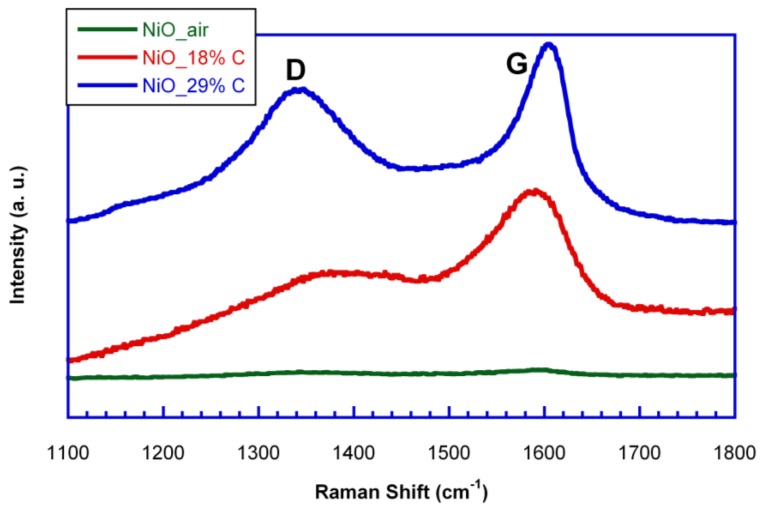
Raman spectra of NiO_air, NiO_18%C and NiO_29%C samples.

In order to study the nature of the in situ generated carbon, Raman spectroscopy measurements were carried out for the samples. Figure 3 shows the Raman spectra of NiO_air, NiO_18%C and NiO_29%C samples. NiO_air sample does not show any band as this material does not contain in situ generated carbon. On the other hand, NiO_18%C and NiO_29%C samples show a typical Raman spectrum of non-graphitic carbons. Both of them show two pronounced peaks, one located at ≈1600 cm^−1^, which corresponds to the G-band, and is ascribed to the E_2g_ graphitic mode. The other band located at ≈1340 cm^−1^, D-band, corresponds to a defect induced mode [27]. Thus, the presence of the D band indicates that the in situ generated carbon is a typically non-graphitizable carbon. In addition, the D band of NiO_18%C sample shows a lower intensity than that corresponding to NiO_29%C sample as it could be expected from the lower amount of carbon that this sample contains. Therefore, in order to see the impact that carbon has on the battery performance, electrochemical measurements were done.

**Figure 4:**
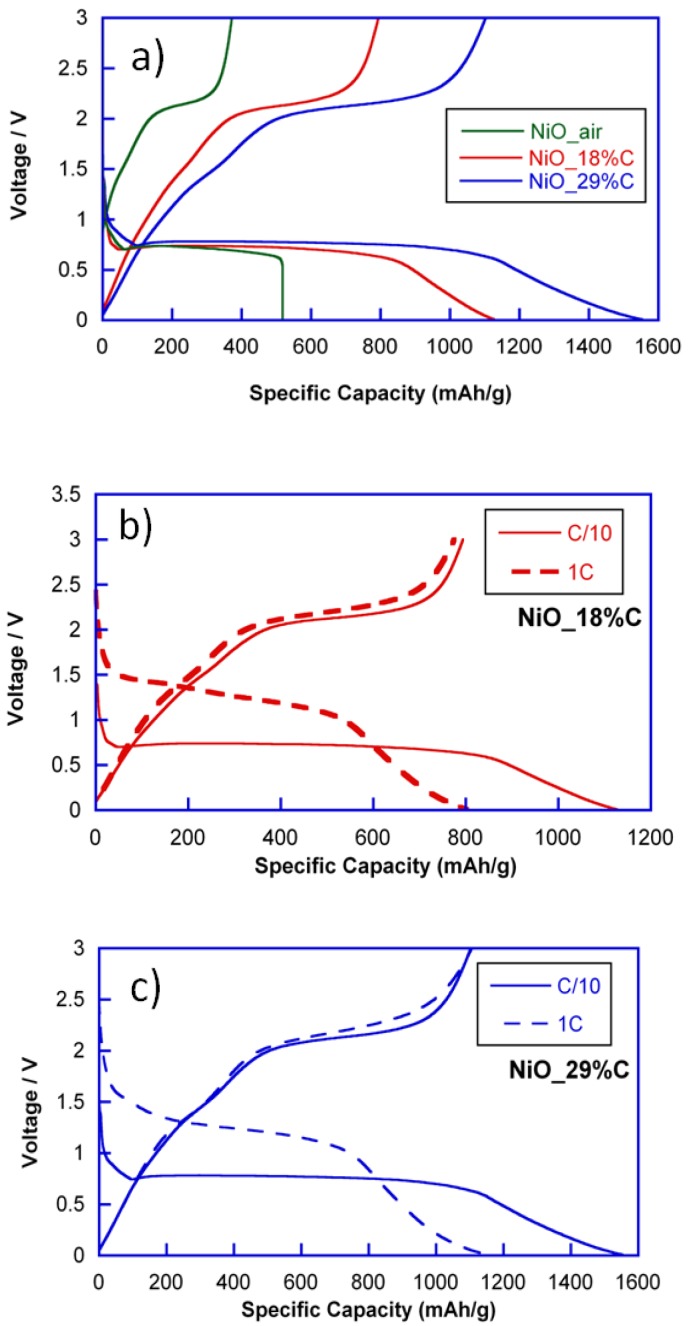
(**a**) First discharge-charge curves for NiO_air, NiO_18%C, and NiO_29%C at C/10, (**b**) comparison of the discharge-charge profiles at C/10 and 1C for NiO_18%C electrode, and (**c**) comparison of the discharge-charge profiles at C/10 and 1C for NiO_29%C electrode.

To evaluate the electrochemical performance, lithium half-cells containing NiO_air and NiO_18%C and NiO_29%C composite materials were discharged at current densities corresponding to C/10 and 1C rates. Figure 4 shows the discharge-charge curves of NiO_air, NiO_18%C and NiO_29%C electrodes at C/10. All of the discharge curves exhibit similar profiles above 0.5 V. The region situated at ≈0.8 V corresponds to the initial reduction of NiO to give Ni and Li_2_O. However, for the composite materials, a gradually sloping low voltage region can be detected, which could be ascribed to the effect of the carbon. NiO_air, NiO_18%C, and NiO_29%C electrodes have delivered initial discharge specific capacities of 518, 1126, and 1552 mAh/g, respectively. On the other hand, all of the charge curves exhibit a plateau that is situated at ≈2 V, which could be attributed to the oxidation of metallic Ni to give NiO. Additionally, the specific capacities of the first charges for NiO_air, NiO_18%C, and NiO_29%C electrodes are 350, 780, and 1150 mAh/g, respectively. In this sense, it is remarkable that there is a capacity loss between the first discharge and charge processes, which could principally be ascribed to the decomposition of the electrolyte and the formation of the Solid Electrolyte Interface (SEI) [11]. 

Due to the electrochemical activity that carbon shows at low voltage regions, the composite with the higher amount of carbon, NiO_29%C, is the one that receives a higher contribution to the capacity that is provided by external factors. However, as it can be deduced from Figure 4a, the length of the plateau located at 0.8 V is larger for NiO_29%C than for NiO_air. This means, that the performance of the conversion reaction is much better for the composites that contain the in situ generated carbon, especially for NiO_29%C. Nevertheless, the amorphous carbon is not the only additional component that these samples contain as they are also composed of metallic nickel particles. In this regard, the presence of metallic nickel could be a key factor when activating the reactivity of the nickel oxide in the first conversion process. The specific capacity of the NiO_air electrode is the smallest one due to the absence of carbon and metallic nickel, together with the heterogeneous morphology of this sample. It is remarkable that the electrochemical behavior of NiO_air decays considerably after a few cycles of charge and discharge. Thus, this sample has not been taken into account for further characterization in this study.

On the other hand, Figure 4b,c show the comparison of the discharge-charge curves for NiO_18%C and NiO_29%C electrodes at C/10 and 1C. As it can be seen, both of the electrodes show a similar tendency. First, an increase in voltage from 0.8 V to 1.5 V can be noticed in the course of the slow speed to the faster speed. Additionally, an irreversible loss of capacity can be perceived, which could principally be ascribed to the decomposition of the electrolyte and to the formation of the SEI. 

**Figure 5:**
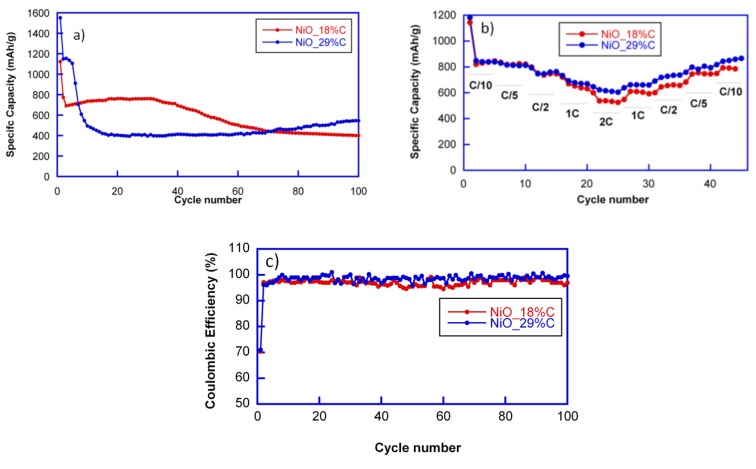
(**a**) Cyclability of NiO_18%C, and NiO_29%C samples at 1C rate and (**b**) rate discharge capabilities for NiO_18%C and NiO_29%C samples, and (**c**) coulombic efficiencies.

The cycling stability of the electrodes was investigated at a current rate of 1C. The capacity loss after the first discharge can be noticed for all cells. However, the cyclability of both of the composites is totally different, as shown in Figure 5a. NiO_18%C electrode maintains a reversible specific capacity of 700 mAh/g during the first 40 cycles. After 100 cycles, the sample still delivers a specific capacity close to 400 mAh/g. This capacity decay could be attributed to the rather heterogeneous morphology of the NiO_18%C sample as well as to the lower amount of the in situ generated carbon matrix in this compound, which improves the connectivity of the particles. These factors may have caused the inactivation of some Li_2_O/Ni regions after discharge due to the disconnection of particles or to the limited access of the electrolyte in the electrode [11]. Meanwhile, the specific capacity of NiO_29%C electrode decays during the first 10 cycles, and after is stabilized at 400 mAh/g up to the 60th cycle. From this cycle on, the specific capacity is increased, providing 550 mAh/g after 100 cycles. This fact could be ascribed to a phenomenon previously observed for other composites with an appreciable amount of carbon: the formation of a polymeric gel that can improve the electrochemical performance [28]. The gel probably contains polyethylene oxide (PEO) oligomers whose formation would be initiated by the reduction of the dimethyl carbonate (DMC) and ethylene carbonate (EC), electrolyte solvents, to lithium alkoxides, and alkylcarbonates. This polymeric gel would coat the particles, possibly contributing to the preservation of their integrity and accumulating additional lithium on its surface, thereby contributing to an extra capacity [29]. Probably, the suitable morphology of the NiO_29%C sample, which provides a larger surface area for an easy ion transport, and the presence of the homogenously distributed carbon matrix, which provides structural stability and facilitates the appearance of this polymeric gel. Thus, these two factors play an important role in the improved cycle and rate performance of NiO_29%C electrode [30,31]. 

The coulombic efficiencies at C/10 (defined as the ratio between charge and discharge capacity) in the first cycles were only of 70% in both of the cases. However, in the successive cycles these values increased noticeably up to 96% and 98%. The highest coulombic efficiency was that of the cell containing NiO_29%C composite. 

The rate performance of the cells was tested at different discharge rates ranging between C/10 and 2C, as shown in Figure 5b. Due to the limited diffusion of lithium ions, the two samples show decreased capacities at the higher rates. Nevertheless, both of the samples deliver 500 mAh/g at 2C. However, NiO_29%C electrode recovered better the capacity when reducing the discharge rate back to C/5 and C/10. As it has been mentioned before, this sample has a smaller particle size, a more homogeneous appearance, and higher carbon and metallic nickel contents. Due to the synergistic effect that these factors could produce, the electrochemical behavior of NiO_29%C is better in all aspects as it has been observed all along the electrochemical characterization. It is remarkable that the capacity values are above 500 mAh/g in both of the cases for the whole range of the different discharge rates, being higher than the theoretical capacity of graphite (375 mAh/g). 

**Figure 6:**
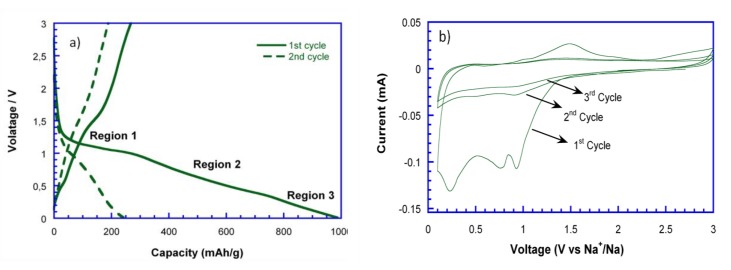

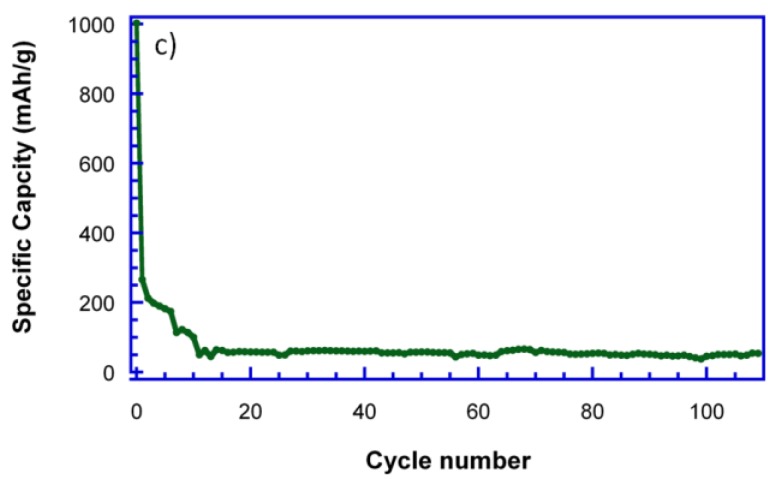
(**a**) Discharge-charge curves, (**b**) cyclic voltammetry (CV), and (**c**) cyclability of NiO_29%C electrode.

As far as we are aware, very little research has been done in the field of NiO anodes for NIBs application up to now. Thus, NiO_29%C composite was selected to test it versus metallic sodium due to its good lithium storage behavior. Figure 6a shows the first two discharge-charge curves of NiO_29%C versus metallic sodium at C/10. As it can be observed, the initial discharge capacity is of 1000 mAh/g, while the charge capacity is of 300 mAh/g. Additionally, the discharge capacity decays to 250 mAh/g in the second cycle. The initial capacity loss could be attributed to several factors, such as the decomposition of the electrolyte and the inactivation of some Na_2_O/Ni regions after discharge due to disconnection. Moreover, the limited amount of Na ions that might be inserted in the carbon matrix, when compared to the storage of Li ions into the carbon, is another important factor that needs to be considered as the radius of Na (r = 0.102 nm) is much bigger than that of Li (r = 0.059 nm) [32]. This fact would also lead to block the diffusion pathways, resulting in a more severe volume expansion and lower kinetics in the conversion reaction. To further explore the electrochemical behavior along with Na-ion uptake/extraction, cyclic voltammetry (CV) was applied for the evaluation of the NiO_29%C anode, as shown in Figure 6b. In this regard, it is important to mention that the galvanostatic tests and CV cannot operate in the same conditions, leading therefore to a variation between the peak position at CV and the regions range of voltage profile. During the 1st sodiation process, three peaks can be observed at 1, 0.8, and 0.25 V, which correspond to the 1st, 2nd, and 3rd regions in the discharge curve, respectively. The first peak could be assigned to the structural destruction of the nickel oxide to ultimately give metallic nickel, Ni° (region1). The second peak located at 0.8 V could be attributed to the insertion of some Na^+^ into the carbon matrix (region 2). The third peak, which corresponds to the gradually sloping voltage low voltage region (region 3) could correspond to a partial decomposition of some electrolytic components. Meanwhile, in the charge process, two peaks can be detected at 1.1 and 1.5 V. The broad peak at 1.1 V shows a very low intensity and could be ascribed to a very limited deintercalation process of Na from the carbon matrix. The peak at 1.5 V is attributed to the oxidation of Ni into NiO. In the subsequent cycles, the reduction and oxidation peaks almost disappear. This fact is also evidenced in the cyclability of the material, Figure 6c, as the capacity drastically decays from the third cycle on. 

In order to investigate the origin of the capacity fade, two sodium half cells containing NiO_29%C electrode were stopped in the third cycle at the charge and discharge configurations at 0.01 and 3 V, respectively. This way, the electrodes were recovered and were used for further characterization. The ex-situ XRD (Supplementary information SI.3). pattern of the discharged electrode showed the diffraction maxima of metallic Ni at 2θ ≈ 45° and 53°, indicating that in the discharged state, NiO is totally reduced to metallic Ni. Moreover, some additional diffraction maxima could be attributed to the presence Na_2_O (77-2148 PDF card), which is the other resulting product from the conversion reaction. Two broad humps at 2θ ≈ 39° and 56° could correspond to NaF (88-2299 PDF card) originating from a possible breakdown of the electrolyte salt NaPF_6_ and PVDF binder. In the XRD pattern that was obtained for the charged cell, only NaF was detected, together with the copper originating from the current collector.

With the purpose of seeing the difference of the metallic nickel content in each electrode, magnetic susceptibility measurements were carried out (Supplementary information SI.4). The discharged electrode showed a higher magnetization than that of the charged electrode for the whole range of the applied magnetic fields. This fact could be assigned to the higher amount of metallic nickel as expected in the discharged state. 

**Figure 7:**
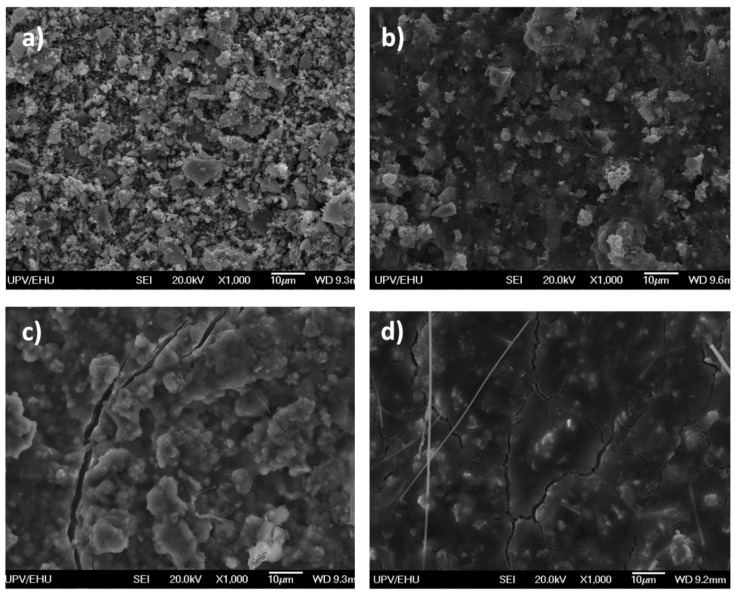
SEM micrographs of NiO_29%C powder (**a**) laminate (**b**) and the discharged (**c**) and charged (**d**) electrodes.

The surface of the electrodes was investigated by SEM characterization. Figure 7a–d show the micrographs corresponding to the initial powder (a), the initial laminate (b), and the discharged (c) and charged (d) electrodes. The initial powder is composed of micrometer size agglomerates that are formed by 5–10 nm average size nanoparticles that homogeneously dispersed in the in situ generated carbon matrix. On the other hand, the initial laminate shows quite a homogeneous aspect, where all of the components of the laminate seem to be uniformly distributed. However, the discharged and charged electrodes show a large difference in their appearances. In this regard, the discharged electrode seems to have some kind of covering layer in the surface besides of showing some fractures. However, the relief of the particles can still be noticed. Meanwhile, this covering layer becomes even thicker for the charged electrode giving place to a polished surface. Moreover, the amount of fractures is notably increased. 

**Figure 8:**
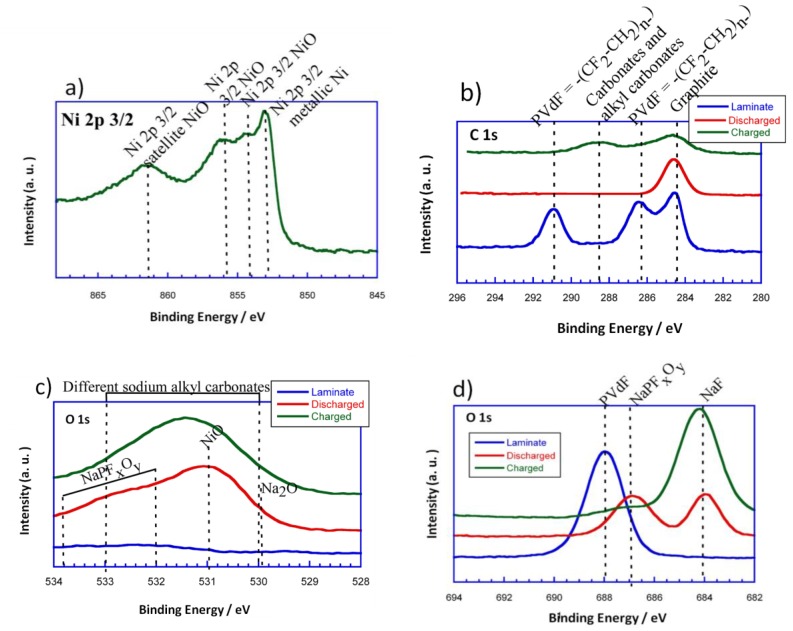
Ni 2p_3/2_ (**a**), C 1s (**b**), O 1s (**c**), F 1s (**d**) photoelectron spectra for the powder, the initial laminate and discharged and charged electrodes.

Thus, in order to see the evolution of surface composition of the initial powder, the initial laminate and the discharged and charged electrodes, X-ray photoelectron spectra (XPS) measurements were carried out. Figure 8a–d shows the high resolution Ni 2p_3/2_, C 1s, O 1s, and F 1s spectra for the initial powder, the initial laminate and the discharged and charged electrodes, respectively. It is important to mention that the Ni 2p_3/2_ spectra corresponding to the laminates is not shown as it is believed not to be reliable due to the interference of F 1s KLL Auger line. In the XPS spectra of Ni 2p_3/2_, there are four peaks centered at 852.8, 853.7, 854.7, and 861.5 eV. The band situated at 852.8 eV could be ascribed to the Ni in the metallic state. The peaks located at 853.7 and 854.7 eV could be attributed to Ni^2+^ in the standard Ni–O octahedral bonding configuration. Finally, the band at 861.5 eV would correspond to the satellite of Ni^2+^ in NiO.

Concerning the evolution of the surface, C 1s is considered to be a very important photoemission line. The C 1s components have been assigned basing on previous XPS studies of carbon base materials [33,34]. This way, the component around 284 eV could be assigned to graphitic-like compounds. As it can be seen, the graphitic signal does not disappear upon discharge. However, the intensity corresponding to this peak is reduced, which means that some kind of SEI or passivation layer has been formed. According to the literature, taking into account that the measurements have been performed using a monochromatic AlK_α_ source, and that after the third cycle we still are able to see the graphitic-like signal, it could be estimated that the thickness of the surface layer that has been formed is smaller than 2.5 nm [35]. Moreover, as the intensity of the graphitic-like signal is reduced, a broad peak can be detected in the range of 288–290 eV for the charged electrode. These signals could be attributed to the appearance of carbonates and alkyl carbonates on the electrode surface when charging. Regarding the binder, the presence of polyvinylidene difluoride (PVdF = −(CF_2_–CH_2_)_n−)_ in the initial laminate is shown by two peaks at 286.5 eV and 291 eV which come from the –CH_2_ and the fluorinated carbon, respectively. However, these signals disappear as the conversion reaction evolves, which points out the vanishing of PVdF from the surface. 

For a better understanding of the surface composition, O 1s (Figure 8b) and F 1s (Figure 8c) XPS measurements were done. As it can be deduced, the O 1s spectrum of the initial powder shows two distinct peaks at 529.4 and 531.6 eV. The peak at 529.4 eV corresponds to the oxygen in NiO, while the one located at 531.6 eV can be ascribed to the shoulder peak of O 1s due to the existence of surface defects [36]. On the other hand, the O 1s spectrum of the initial laminate does not show any signal. This makes sense as the NiO particles are homogeneously dispersed with all of the components of the laminate. The discharged electrode has a dominating component at 531 eV that corresponds to the NiO particles that have not been reduced to Ni (0) due to their disconnection. The main signal extends to lower and higher binging energies, 529.7 eV and 532–534 eV, respectively. The signal that is located at 529.7 eV could be attributed to Na_2_O. This is in good agreement with the evolution of the conversion reaction as metallic Ni particles would be homogenously distributed in a sodium oxide matrix. The broad shoulder that is found at 532–534 eV could be assigned to the presence of NaPF_x_O_y_, due to the partial decomposition of the electrolyte (NaPF_6_). On the other hand, the charged electrode shows a wide signal in the rage of 530–533 eV. This signal could be attributed to the different sodium alkyl carbonates that have been formed in the surface of the electrode.

In the F 1s photoelectron line, a pronounced peak appears for the initial laminate at 688 eV, which corresponds to the presence of PVdF. The discharged electrode, for its part, shows two peaks at 684 and 687 eV that could be attributed to the formation of NaF and NaPF_x_O_y_, respectively, due to partial decomposition of the electrolyte as it has been previously reported for Li-ion batteries [37]. Meanwhile, a dominating component situated at 684 eV and a small shoulder at 687 eV can be identified for the charged electrode. These peaks would correspond in this case too, to NaF and NaPF_x_O_y_, respectively. However, the intensity of the peak corresponding to NaF is higher in the charged electrode than in the discharged one. Therefore, it could be said that the degree of decomposition of the electrolyte is increased with the evolution of the cycling. 

In order to further confirm the aforementioned results, Fourier transform infrared (FTIR) spectra of both of the electrodes were measured, as shown in (Supplementary information SI.5). In both the discharged and charged electrodes some peaks corresponding to vibration of C–C sp^2^ and sp^2^/sp^3^ bonds (located at 850 and 1350–1450 cm^−1^) were detected, which could be mainly attributed to the “in situ” generated and additive carbons. Moreover, both of the spectra showed the signal of NaF at 550 cm^−1^. Additionally, in both of the cases, a group of peaks that were located in the wide range of 1000–1100 cm^−1^ wavelenghts, was present. These signals could be ascribed to the presence of: (1) PO_4_ or PF_x_O_y_ stretching due to some decomposition product of the electrolyte, (2) C–C bonds which would indicate the existence of different long-chain alkyl groups, and (3) C–O bonds due to the presence of carbonates. However, the charged electrode showed some additional peaks (mainly at 1550–1600 cm^−1^) that were not present in the discharged electrode. These peaks corresponded to the presence of NaCO_3_R [38]. Therefore, these results confirm the existence of sodium alkyl carbonate compounds in the surface of the electrode in the charged state, while in the discharged state, a layer of reduced carbon would be covering the surface of the electrode. 

When taking into account all of the results that have been obtained up to the moment from the post mortem analysis of the electrodes, it could be suspected that after the third discharge all of the carbon constituting the sample (the in situ generated one plus the ketjen) is totally reduced . This way, the covering layer detected by SEM characterization could be assigned to the reduced carbon that somehow, would be surrounding the active material particles. This is in good agreement with the result obtained by the XPS study of C 1s, as the only peak that is detected in the discharged electrode is assigned to graphitic-like compounds. Meanwhile, the conversion reaction would be taking place in those areas that have not been disconnected yet, giving place to Ni (0) and Na_2_O. Nevertheless, some remaining NiO would be left due to the presence of some inactive areas in the electrode. In the subsequent charge, the carbon on the surface would react with the organic components that constitute the electrode, such as the electrolyte solvents, in order to be oxidized and give place to different sodium alkyl carbonates, NaCO_3_R. Indeed, the formation of these carbonates would give a polished aspect to the surface of the charged electrode as it has been previously observed in the SEM micrograph. Due to the large volume expansion that this reaction implies, the electrode surface would be subjected to several mechanical stresses during this period. As a result, the surface of the electrode would be fractured and some crackings would appear, as it has been demonstrated by SEM characterization. The reversibility of the surface reaction and the small thickness of the layer (<2.5 nm) corroborate the unstable nature of the SEI or the passivation layer that is formed. Consequently, the NIB with the NiO/C composite reveals a poor cycling stability.

## 3. Materials and Methods

### 3.1. Materials and Reagents

The following materials and reagents were used as purchased without further purification: citric acid monohydrate (99.5%, Sigma-Aldrich, St. Louis, MI, USA) and nickel (II) hexahydrate (98%, Sigma-Aldrich). 

### 3.2. Sample Preparation

Three nickel oxide samples were synthesized by the freeze-drying method. For the sample designated NiO_air only Ni(NO_3_)_2_·6H_2_O was dissolved in 25 mL of water. For the other two samples C_6_H_8_O_7_·H_2_O and Ni(NO_3_)_2_·6H_2_O reagents were added in the molar ratios of 0.25:1 and 1:1, in order to produce composites with different carbon contents. The resulting solutions were subsequently frozen in a round-bottom flask that contained liquid nitrogen. Afterwards, the round bottom flasks were connected to the freeze-dryer for 48 h at a pressure of 3 × 10^−1^ mbar and a temperature of −80 °C to sublime the solvent. The as-obtained precursors were subjected to a single heat treatment at 400 °C for 6 h. The heat treatment of the NiO_air sample was carried out in air while the other two samples were calcined in a nitrogen atmosphere. Subsequently, the products were ball-milled for 30 min. 

### 3.3. Characterization

A Perkin-Elmer 2400CHN analyzer (St. Louis, MI, USA) was employed to determine the carbon content of the samples. Structural characterization of the samples was carried out using X-ray powder diffraction with a Bruker D8 (Billerica, MA, USA) Advance Vario diffractometer using CuK_α_ radiation. The obtained diffractograms were profile-fitted using the FullProf program [39]. The morphologies of the materials were studied by Transmission Electron Microscopy (TEM) using a FEI TECNAI F30 (Hillsboro, OR, USA) and by a scanning electron microscope (JEOL JSM 7500F (Akishima, Tokyo, Japan)). Magnetic susceptibility measurements (dc) were carried out at 300K with a Quantum Design SQUID magnetometer. X-ray photoelectron spectra were (XPS) were obtained on a SPECS system that was equipped with a Phoibos 150 1D-DLD analyzer (Berlin, Germany) and a monochromatic AlK_α_ (1486.6 eV) source. Raman spectroscopy was carried out using a InVia Raman spectrometer (Wotton-under-Edge, United Kingdom) using Ar^+^ laser excitation with a wavelength of 514 nm.

To evaluate the electrochemical performance 2032 coin cells were assembled to evaluate the electrochemical performances of the samples. To prepare the electrodes, the active materials were mixed with conducting carbon black (Super P, Timcal) and polyvinylidene fluoride (PVDF) binder, with weight ratios of 70:15:15 and was dispersed in N-methyl-2-pyrrolidone (NMP) to form a slurry. The slurry was then cast onto Cu current collectors and dried at 120 °C in a vacuum oven overnight. For the lithium ion batteries, electrochemical cells with metallic lithium foils as counter electrodes, Celgard 2400 polypropylene separators (Charlotte, NC, USA) and 1 M LiPF_6_ in 50–50% ethyl carbonate (EC) and dimethyl carbonate (DMC) as the electrolytic solution, were assembled in an Ar-filled glove box. For the sodium ion batteries, metallic sodium foils were used as counter electrodes. The electrolyte was 1 M NaPF_6_ in 50–50% ethyl carbonate (EC) and dimethyl carbonate (DMC) solution, with 1 wt % FEC All of the electrochemical and electrochemical measurements were carried out on a Bio-Logic VMP3 potentiostat/galvanostat (Seyssinet-Pariset, France) at room temperature. Typical electrode loadings were 1.3 mg/cm^2^. 

CV was performed in a potential window of 0.01-3 V with a scanning rate of 5 mV for three cycles. The galvanostatic charge/discharge experiments were performed between 0.01 and 3 V at 0.1C and 1C current rates where 1C is defined as the rate at which the total theoretical capacity based on full conversion can be discharged in one hour:

NiO + 2A^+^ + 2e^−^ ↔ Ni + A_2_O (where A = Li or Na)

To calculate the capacity values, the weight of only the NiO was considered, not including the in situ carbon. For the post-mortem analysis of the Na configuration batteries, cells were opened in an Ar-filled glove box. After, the electrodes were washed during a few seconds three times with a large excess of dimethyl carbonate (DMC) in order to dissolve the residual salt of the electrolyte that could stay in the surface of electrodes. The harvested electrodes were used for further analysis, such as XRD, SEM, FTIR, and magnetic measurements.

## 4. Conclusions

NiO_air, NiO_18%C, and NiO_29%C samples were successfully prepared by a freeze-drying method. X ray diffraction measurements for NiO_air sample showed that all of the diffraction peaks could be indexed to nickel oxide. For NiO_18%C and NiO_29%C, metallic nickel was detected as well as nickel oxide. The morphologic study demonstrated the heterogeneity of NiO_air sample, with an average particle size of 5–50 nm. However, NiO_18%C and NiO_29%C are more homogeneous, have smaller particle size and are present an in situ generated amorphous carbon matrix. The most significant result was the reduction of particle size with the increasing of carbon amount. Magnetic measurements allowed calculating the amount of metallic nickel for each sample. NiO_air, NiO_18%C, and NiO_29%C electrodes were employed in LIBs and NiO_29%C electrode was the one with the highest specific capacity, best cycleability, highest coulombic efficiency, and best rate discharge capability. This fact could be ascribed to the higher amount of metallic nickel and carbon, the smaller particle size and the homogeneous character that this sample has in comparison to the other materials. On the other hand, the NIB with the NiO_29%C electrode revealed a poor cycling stability. Post-mortem analyses (ex.situ XRD, SEM, magnetic measurements, XPS, and FTIR) revealed that this fact could be mainly ascribed to the absence of a stable SEI upon cycling. In this regard, the surface reaction that occurs when discharging (reduced carbon) and charging (NaCO_3_R) the electrode, implies a huge volume expansion, causing the fracture of the electrode, and leading, therefore, to a poor electrochemical performance of the NIB. Additionally, the large amount of carbon that NiO_29%C electrode contains is another important factor to be considered since the storage of Na into carbon is very limited. Consequently, the diffusion pathways could be blocked, promoting the deterioration of the kinetics of the conversion reaction.

## Figures and Tables

**Table 1:** Percentage of carbon in each sample.

Sample	% C
NiO_air	0
NiO_18%C	17.1 (8)
NiO_29%C	28.9 (5)

**Table 2:** Theoretical and experimental cell parameters for NiO_air, NiO_18%C, and NiO_29%C compounds.

Sample	Experimental Cell Parameters (Å) a = b = c	Theoretical Cell Parameters (Å) a = b = c	Space Group
NiO___air	4.1783 (4)	4.177	Fm-3m
NiO___18%C	4.1759 (2)		
NiO___29%C	4.175 (2)		

**Table 3:** Values of the saturation magnetization and the percentages of metallic nickel for each sample.

Sample	Saturation Magnetization (emu/g)	Percentage of Metallic Nickel (%)
NiO___air	0.21	<1
NiO___18%C	2.87	5
NiO___29%C	22.6	41

## Supplementary Materials

The following are available online at www.mdpi.com/2079-4991/7/12/423/s1, Figure S1: SEM images of (a) NiO_air, (b) NiO_18%C and (c) NiO_29%C samples, Figure S2: Hysteresis loops at room temperature of the NiO_air, NiO_18%C and NiO_29%C samples, Figure S3: Ex-situ XRD patterns of the NiO_29%C sample and the discharged and charged electrodes, Figure S4: Hysteresis loops at room temperature of the discharged and charged electrodes and Figure S5: FTIR spectra of the discharged and charged electrodes.
