# Supplementary material for: Physico-Chemical and Electrochemical Properties of Nanoparticulate NiO/C Composites for High Performance Lithium and Sodium Ion Battery Anodes
Source: Nanomaterials (Basel). 2017 Dec 2;7(12):423. doi: 10.3390/nano7120423 (PMC5746913; doi:10.3390/nano7120423)
Supplement: Supplementary file 1 [file nanomaterials-07-00423-s001.zip › Supplementary information/Supplementary Information.docx]

| 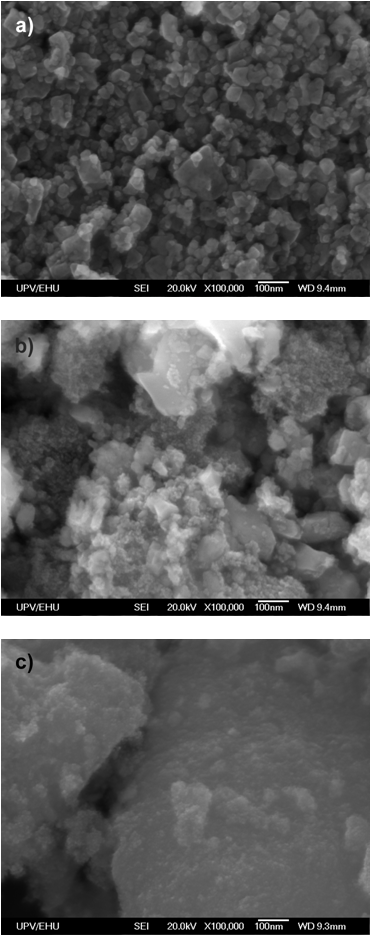 |
| --- |
| **SI. 1.** SEM images of a) NiO_air, b) NiO_18%C and c) NiO_29%C samples |

| 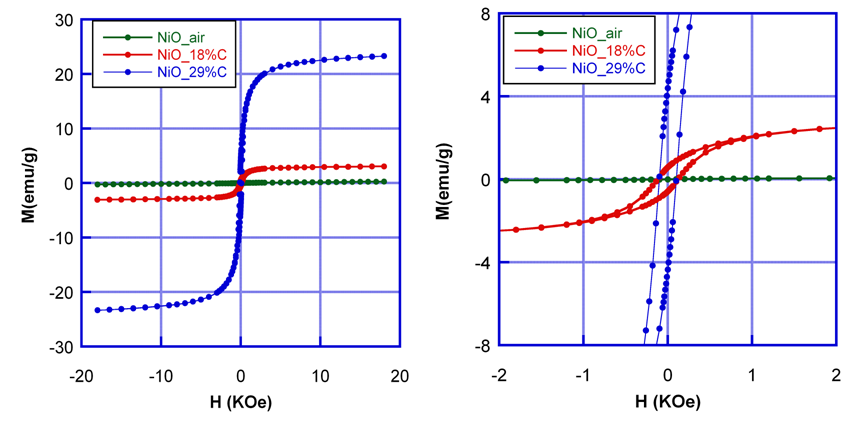 |
| --- |
| **SI. 2.** Hysteresis loops at room temperature of the NiO_air, NiO_18%C and NiO_29%C samples |

| 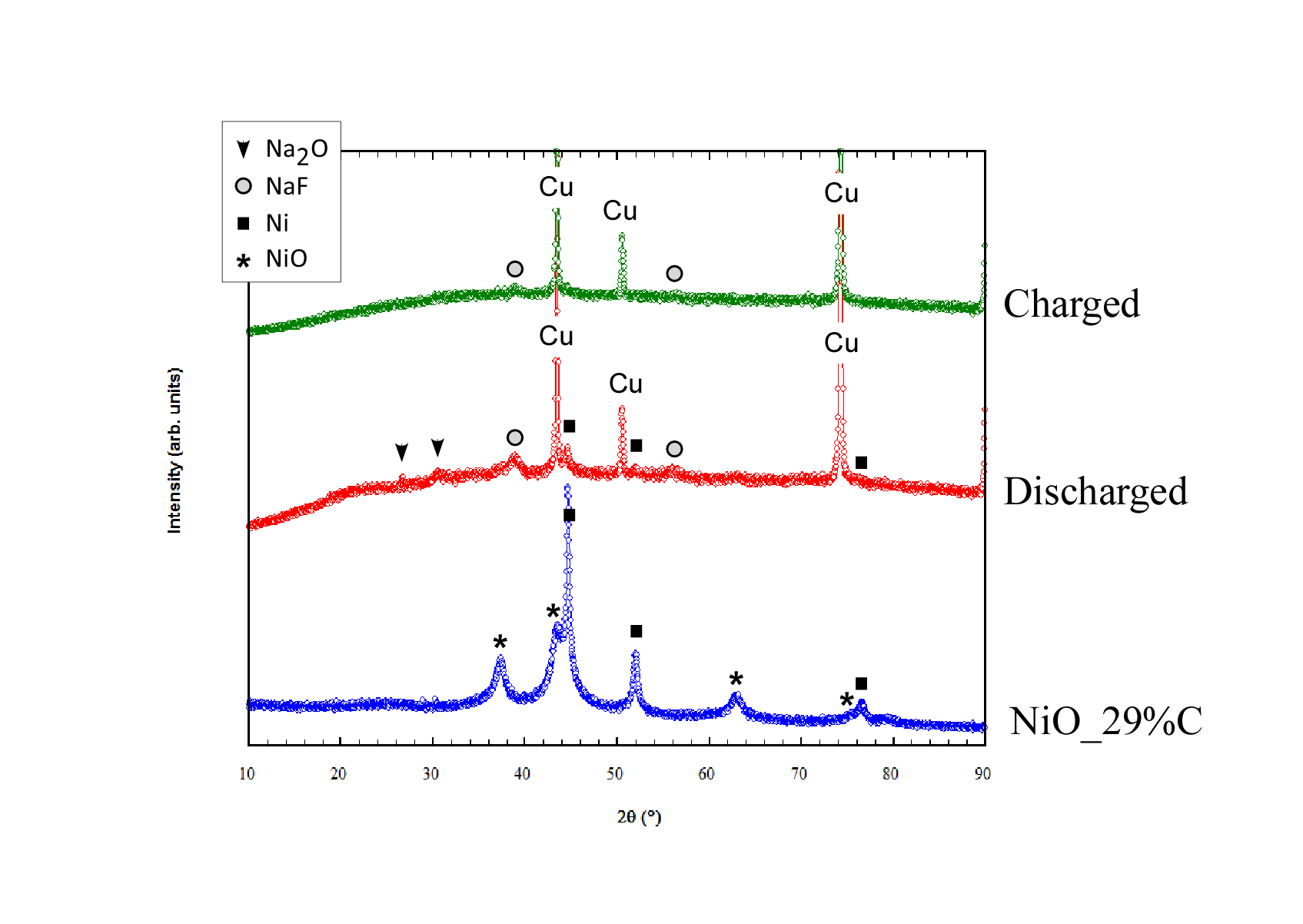 |
| --- |
| **SI. 3.** Ex-situ XRD patterns of the NiO_29%C sample and the discharged and charged electrodes |

| 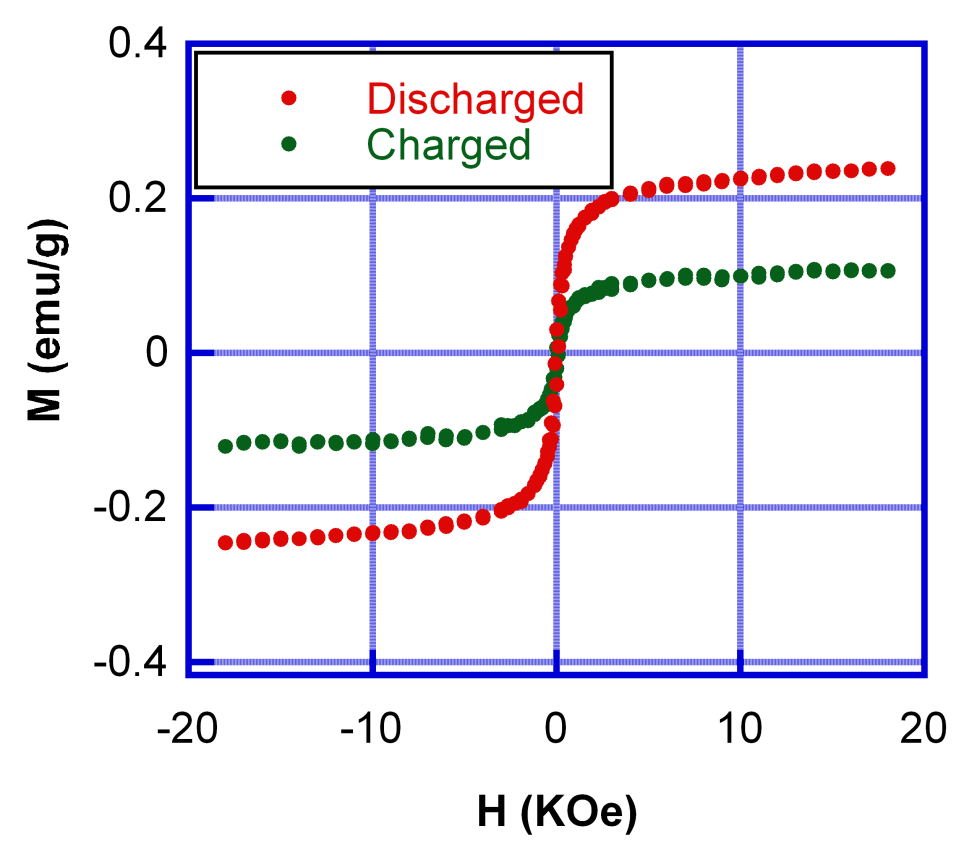 |
| --- |
| **S. 4.** Hysteresis loops at room temperature of the discharged and charged electrodes |

| 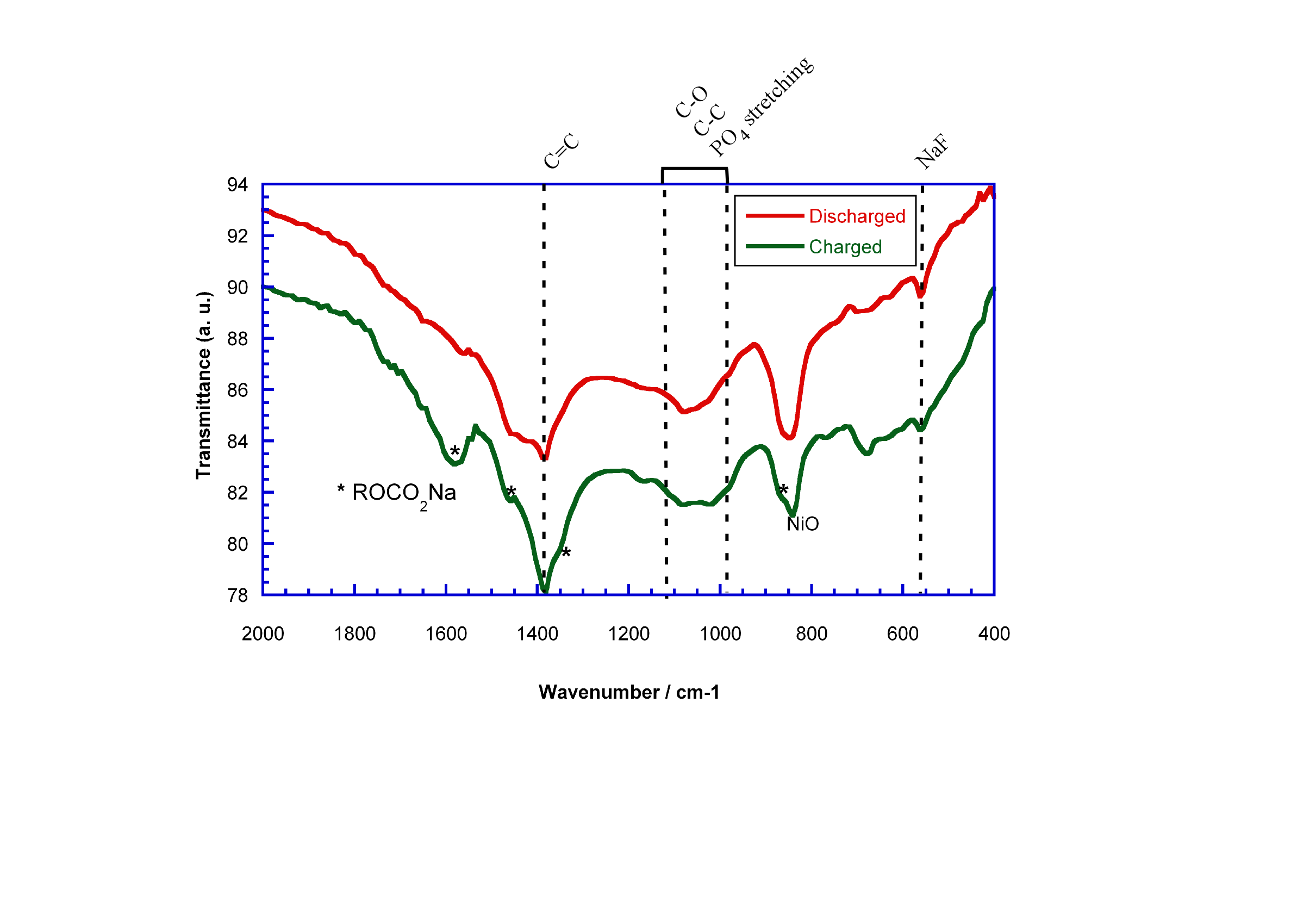 |
| --- |
| **SI. 5.** FTIR spectra of the discharged and charged electrodes |
