# Supplementary figures and images for: Physico-Chemical and Electrochemical Properties of Nanoparticulate NiO/C Composites for High Performance Lithium and Sodium Ion Battery Anodes
Source: Nanomaterials (Basel). 2017 Dec 2;7(12):423. doi: 10.3390/nano7120423 (PMC5746913; doi:10.3390/nano7120423)

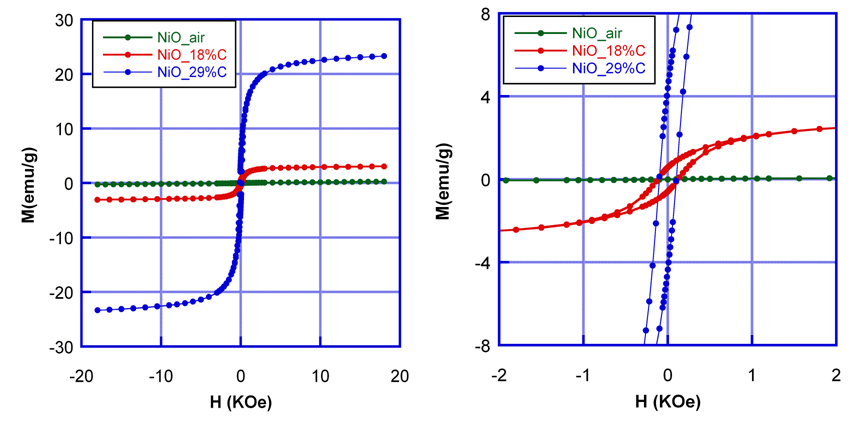

Supplement: Supplementary file 1 [file nanomaterials-07-00423-s001.zip › Supplementary information/SI. 2.tif]

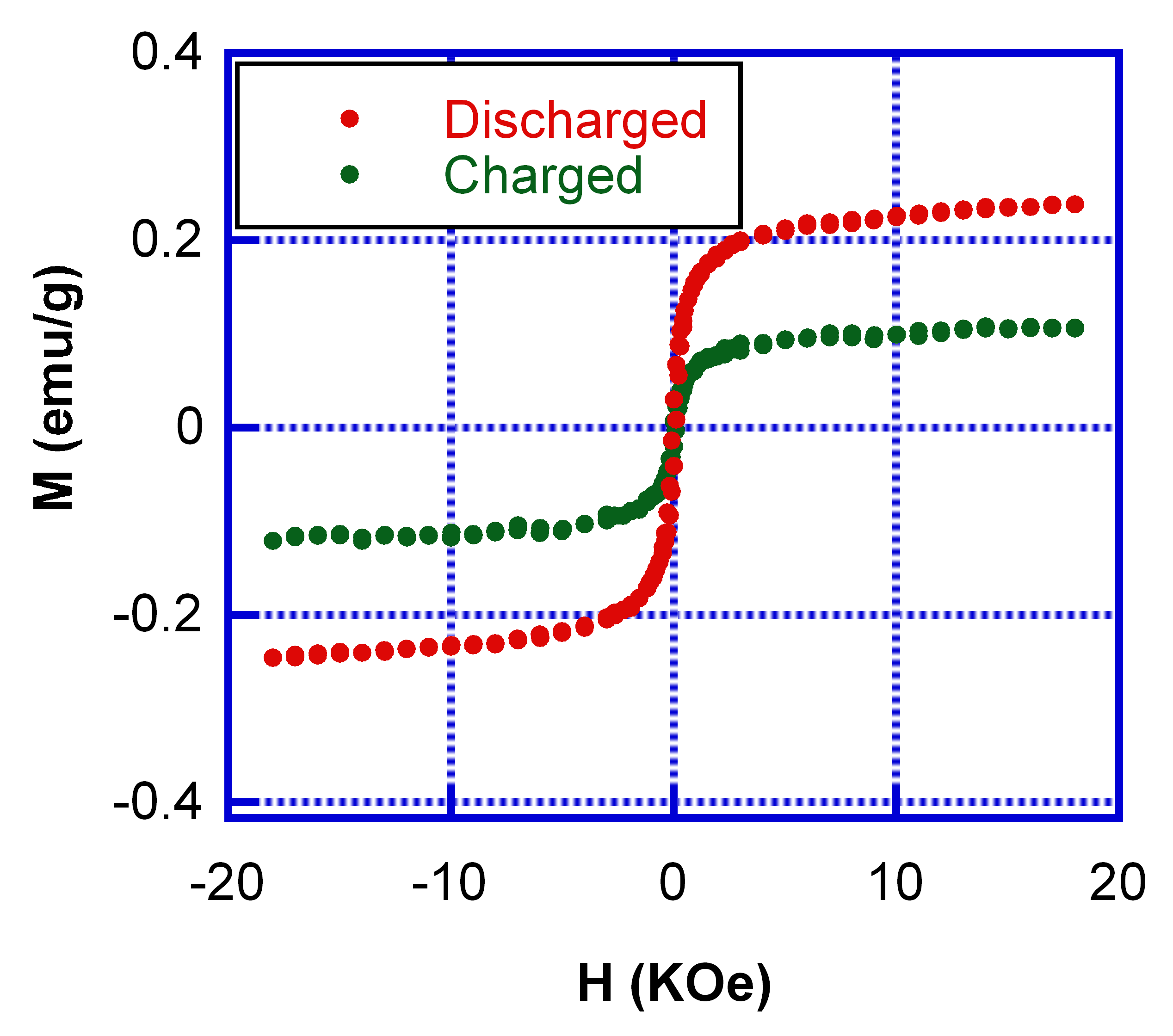

Supplement: Supplementary file 1 [file nanomaterials-07-00423-s001.zip › Supplementary information/SI. 4.TIF]

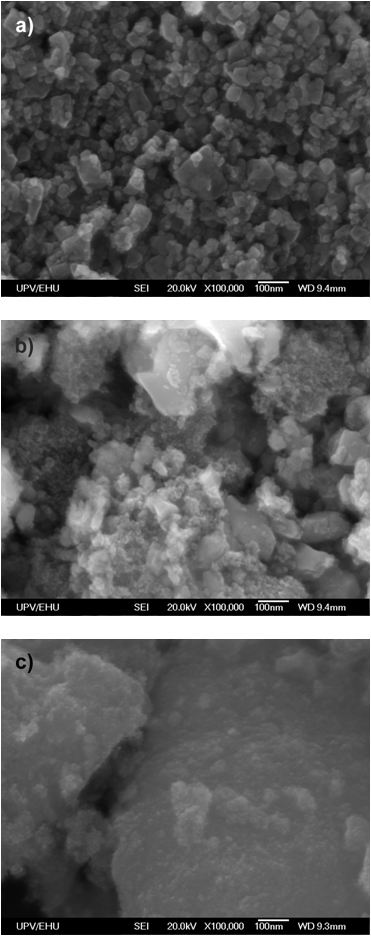

Supplement: Supplementary file 1 [file nanomaterials-07-00423-s001.zip › Supplementary information/SI.1.tif]

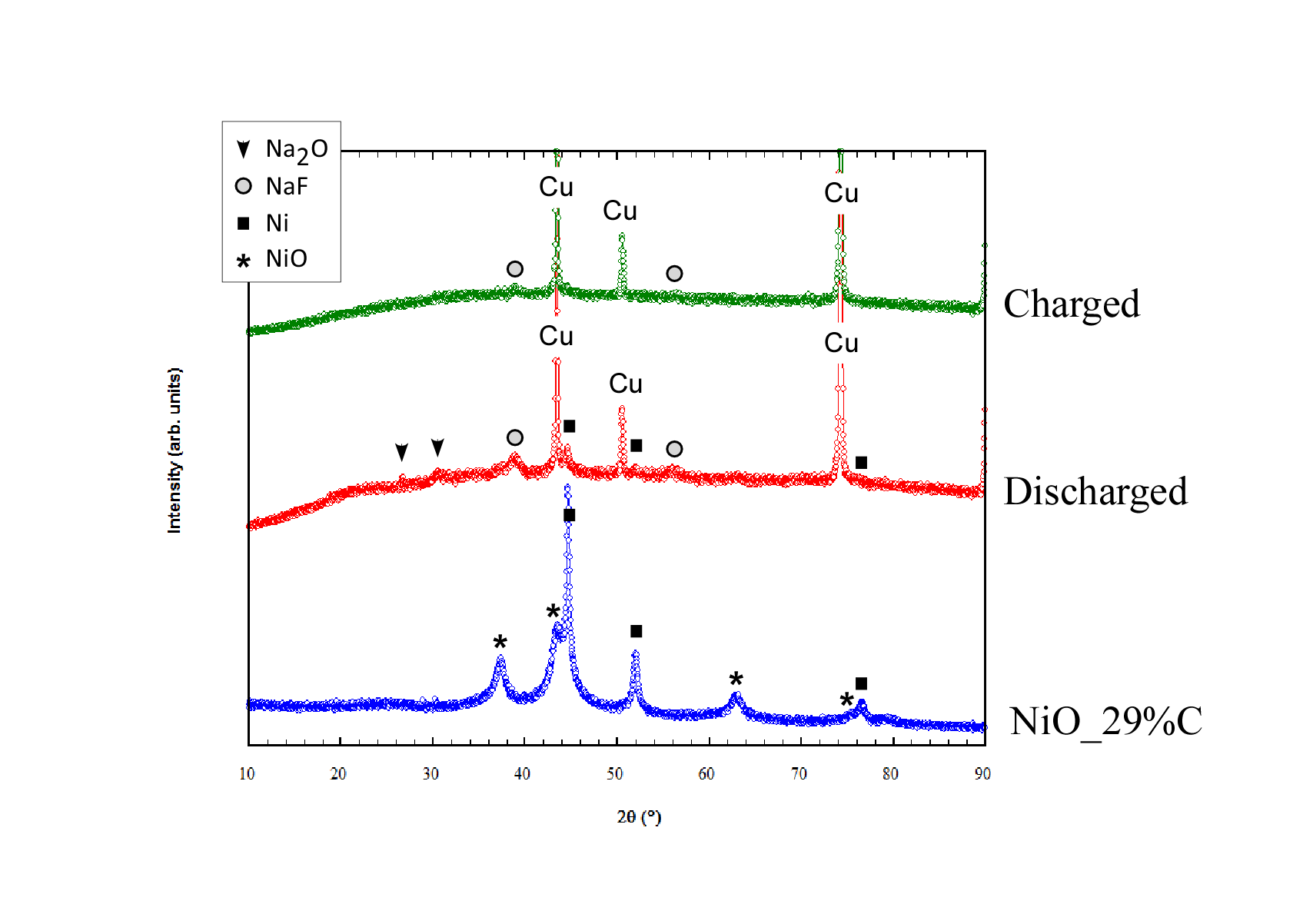

Supplement: Supplementary file 1 [file nanomaterials-07-00423-s001.zip › Supplementary information/SI.3.tif]

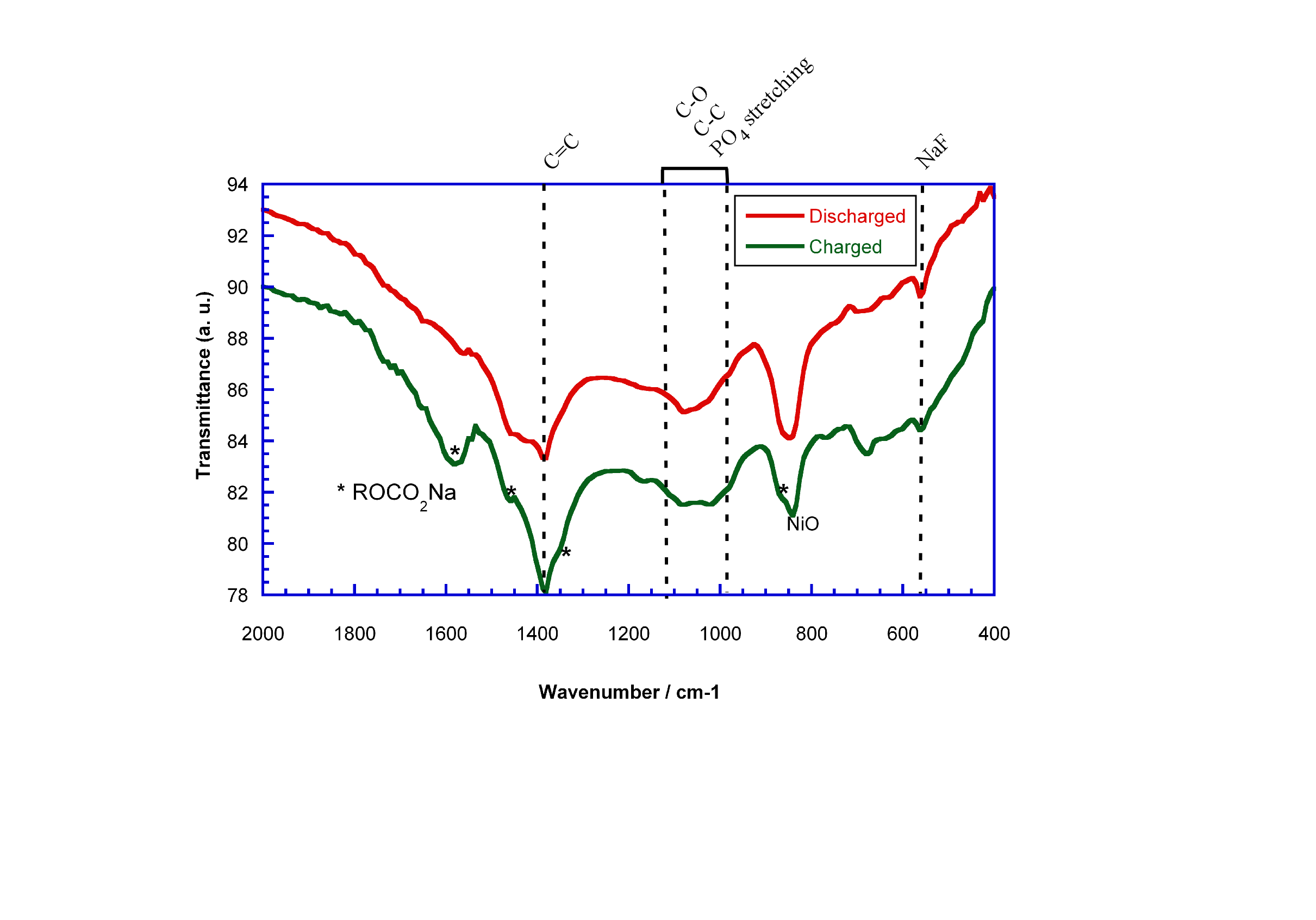

Supplement: Supplementary file 1 [file nanomaterials-07-00423-s001.zip › Supplementary information/SI.5.tif]
